# The Application of Metabolomics in Forensic Science with Focus on Forensic Toxicology and Time-of-Death Estimation

**DOI:** 10.3390/metabo11120801

**Published:** 2021-11-26

**Authors:** Joanna Dawidowska, Marta Krzyżanowska, Michał Jan Markuszewski, Michał Kaliszan

**Affiliations:** 1 Department of Biopharmaceutics and Pharmacodynamics, Medical University of Gdańsk, 80-210 Gdańsk, Poland; jo.dawidowska@gumed.edu.pl (J.D.); markusz@gumed.edu.pl (M.J.M.); 2 Department of Forensic Medicine, Medical University of Gdańsk, 80-210 Gdańsk, Poland; marta.krzyzanowska@gumed.edu.pl

**Keywords:** metabolomics, forensics, biomarker, postmortem interval (PMI), thanatometabolomics

## Abstract

Recently, the diagnostic methods used by scientists in forensic examinations have enormously expanded. Metabolomics provides an important contribution to analytical method development. The main purpose of this review was to investigate and summarize the most recent applications of metabolomics in forensic science. The primary research method was an extensive review of available international literature in PubMed. The keywords “forensic” and “metabolomics” were used as search criteria for the PubMed database scan. Most authors emphasized the analysis of different biological sample types using chromatography methods. The presented review is a summary of recently published implementations of metabolomics in forensic science and types of biological material used and techniques applied. Possible opportunities for valuable metabolomics’ applications are discussed to emphasize the essential necessities resulting in numerous nontargeted metabolomics’ assays.

## 1. Introduction

When discussing forensic science, its multidisciplinary nature must be addressed. Over the past years, diagnostic methods have flourished. However, the ongoing need for highly specific and highly sensitive analytical methods continues to increase very rapidly. Providing accurate results is crucial when considering kinship testing, cause of death determination, and the pronouncement of guilt, for example. Metabolic alterations that occur in an organism are suggested to be highly valuable in these cases. The tremendous volume of data obtained during profiling of a cell’s metabolome requires comprehensive and sophisticated analytical strategies [1].

The immense and promising potential of metabolomics has already been demonstrated in the framework of drug toxicity studies, differential diagnostics of diseases, and metabolomic profile modeling. Deeper investigation into the metabolic pathways and the functionality of the human organism may lead to a better understanding of the functioning of the body and permeable applications of metabolomics in clinical medicine in the future. Sample pretreatment and preparation must be considered, as the chemical and physical properties of postmortem material might be marginally but significantly divergent. Recently, a study by Akcan et al. [2] investigated the markedly increasing popularity of -omics among medico-legal specialists. Through a comprehensive review of the available literature, they drew a conclusion about the beneficial impact of -omics, especially when more than one approach can be applied. Nevertheless, metabolomics has great potential for the identification and characterization of potential biomarkers of psychoactive substance and drug abuse. With many newly synthesized compounds appearing on the market every year, rapid metabolomic analyses offer hope for faster and more accurate patient help and problem solving, as described in the comprehensive work by Szeremeta et al. [3].

## 2. Metabolomics and Its Branch: Thanatometabolomics

Metabolomics is considered to be the youngest of the “-omics”, including genomics, proteomics, and transcriptomics, and focuses on low-molecular-weight products (<1 kDa) that are present within single cells, tissues, or whole organisms and are useful for describing interactions between different physiological aspects and pharmacokinetic processes while appraising the condition of the organism [4,5,6,7]. The broad field of -omics’ science aims to qualitatively and quantitatively analyze the small-molecule products found in biological samples, the amount and composition of which may vary depending on environmental factors, genetic modifications, and pharmacological substances [8,9].

Because of the complexity and huge diversity of areas involving ‘-omics’, they can be divided into smaller, simpler subdisciplines that focus on a particular branch of science.

Compared with other experimental methods, metabolomics appears to be a new, reasonable, and cost-effective tool in forensic analysis: (1) More diverse biological samples can be analyzed, (2) the cost of analysis per sample is lower than that of other -omics, and (3) the assays are relatively less time consuming. Furthermore, Castillo-Peinado et al. [10] emphasized the impact of the inevitably developing aspect of the growing capacity of the data provided using multiple instruments.

### 2.1. Different Metabolomics’ Strategies

The specific strategy for a metabolomics’ study requires miscellaneous types of initial information, which is determined by the choice between targeted analysis (demonstrated by qualitative and quantitative results) [11], metabolomics’ profiling [12], fingerprinting, or footprinting. Each of the approaches demands different practical procedures depending on the essential purpose of the assay [13]. A prospective view of sample diversity will constitute a promising field considering sufficient laboratory work organization.

### 2.2. Thanatometabolomics

Thanatometabolomics is considered a promising research approach in forensic science. By expanding the number of small molecules that might be identified and quantified in an analyzed biological sample, the pharmacokinetics, pharmacodynamics, and the mechanisms regularly occurring in the organism after taking a prescribed medication or consuming ethyl alcohol can be verified. Toxicogenetics is considered to have crucial significance in forensic examinations and may provide valuable information about genotypes, phenotypes, and possible specific alterations [14,15,16].

Moreover, metabolomics can be used to identify potential biomarkers for numerous diseases. Influencing the concentrations of certain molecules can lead to life-threatening consequences and eventually death. Metabolomics, with advanced analytical techniques, such as separation techniques combined with mass spectrometry or magnetic resonance, has already been demonstrated to be a valuable source of information about the pathophysiology of various health conditions, including mental disorders [17]. Pang et al. [18] presented a review highlighting the great importance of metabolomics in the future of clinical pharmacology, hoping for rapid and significant development in this field.

In addition, the metabolomics’ approach as a tool to determine postmortem interval variations within an organism’s metabolome is nowadays of great interest to many researchers. As traditional methods are not sufficient in obtaining trustworthy results because of biological fluctuations that take place in the organism, metabolomics shows great potential in this field of science [19].

## 3. Goals of Metabolomics’ Assays

Metabolomics provides an appropriate source of data for metabolic alterations under various conditions. The results might affect the assessment of a human’s state of health, bacterial microflora, and changes caused by intoxication. Nevertheless, every stage of analysis must be properly designed, and accurate conditions should be used during measurements; for example, examining the same sample might provide varying results on different days [20]. Metabolomics could be used for more appropriate forensic expertise worldwide.

### 3.1. Postmortem Interval

The postmortem interval (PMI) is the time between death and the discovery of the body. The PMI is extremely important in criminal investigations and has been an object of research for many years. Inaccurate or incorrect estimations of the PMI can mislead an investigation and result in an unjust court judgment. While conducting an analysis focused on postmortem metabolomic profile alterations, there is a need to confront a test group with a reliable control group, in order to avoid misleading conclusions [21]. The alterations within the body that occur postmortem differ from case to case, as each case entails its own unique circumstances. Generally, techniques used in PMI estimations require information about body temperature fluctuation, rigor mortis, body mass, body coverings, and other environmental conditions, which are often difficult to correctly estimate [22,23,24]. However, as already ascertained, most attempts to determine the time of death are ineffective due to complex postmortem processes, which increase with time. More importantly, Henssge et al. [24] emphasized the fundamental need for control case studies to obtain reliable and comparative results. When the research object is a skeleton, an immense spectrum of methods is available, for example, measurement of the levels of specific genomic DNA degradation and the amount of bone proteins, triglycerides, and cholesterol. However, these features are strongly influenced by environmental conditions, and interpreting their results may not be accurate. Other possibilities include methods in which radiocarbon or strontium dating is performed [25,26]. As already mentioned by Buchan et al. [25], the suitability of radiocarbon dating and its utility in sample dating might provide insight into temporal groups. Overall, handling corpses that have not yet undergone putrefaction and autolytic processes requires different procedures other than collecting samples from skeletons. Methods available for nondegraded sampling have not sufficiently evolved, which is the reason why the metabolomics’ approach is highly promising. Thus far, extensively examined human urine samples constitute an encouraging assay material for metabolic phenotyping [27], potentially providing scientists with answers to recurrent questions such as the cause of death based on the concentrations of several biomarkers, which are considered practical and objective indicators [28], and cellular changes that occur at the moment of death with regard to PMI estimation. Pesko et al. [29] presented a study in which a human tissue and a rat postmortem tissue were examined and compared, looking for possible PMI markers, pointing out threonine, tyrosine, and lysine show the greatest potential for being one. Another six metabolites indicated by Jawor et al. [30] increased their levels in plasma and urine with increasing PMI among a group of stillborn calves.

Dai et al. [31] presented a study in which a total of 39 metabolites were found to be associated with PMI. Then, the following combinations of various numbers of metabolites were used to establish support vector regression (SVR) models to investigate the PMI, proving that metabolomics has a great potential in determining PMI of poisoned rats. In contrast, Aronson et al. [32] advocated the significance of metabolomics in the process of conducting an assessment without full awareness of underlying mechanisms.

### 3.2. Toxicology

Toxicological analysis proceeds with a regular work scheme in regard to determining the cause of acute or lethal intoxication. Due to the potential of metabolomics for determining the presence of specific molecules in an examined sample, this approach is becoming increasingly popular in regular work. The uncertainty of the dose and the time of intake remain invariable factors. As an alternative to standard forensic toxicology methods, the metabolomics’ approach can allow confirmation of drug consumption or manipulation attempts. What is more, metabolomics can be a promising tool in discovery and understanding of the mechanism of action of new narcotic drugs’ chemicals.

#### 3.2.1. Drugs

The problems of excessive drug consumption and overdosing are constantly increasing in modern society. New psychoactive substances (NPS) are also of significance, as there is scarcely any information on their mechanism of action or side effects available. Metabolomic studies offer the chance to get quicker and more precise answers to the questions related to drugs of abuse problems [33]. According to the literature, chronic and acute administration of toxic substances leaves a footprint on the body’s physiological fluid composition. Among addictive substances, heroin, morphine, codeine, and products of their metabolism are speculated to have the principal impact on the organism’s condition. Most importantly, the toxins’ intermediate metabolites are usually measured by the metabolomics’ approach rather than the prodrug itself.

Gamma-hydroxybutyric acid, known as GHB, is commonly referred to as a ‘club drug’. Its short detection window time and the differentiation between levels of endogenous or exogenous origin make the process of analysis more complex and challenging. Steuer et al. [34] processed a sudy of untargeted metabolome approach in order to find new biomarkers of GHB, having determined three new GHB metabolites, which can be treated as GHB intake biomarkers if confirmed by further studies. 

Heroin is strongly considered a prodrug, and numerous assays involve animal experimental models, i.e., in rats and mice, to verify the strength and intensity of the influence of certain metabolites [35,36]. These metabolites readily cross the blood–brain barrier and influence the central and peripheral nervous systems. During peripheral administration of morphine, 6-monoacetylmorphine (6-MAM) and morphine-6-glucuronide, which are heroin metabolites, influence the brain and exert the intoxicating effect of heroin. Way et al. [37] expected that both morphine and 6-MAM have similar significance within heroin’s impact on the organism, but more recent research conducted by Andersen et al. [38] points out and advocates for the superiority of 6-MAM, which was credited with stronger behavioral effects. Furthermore, as emphasized by Gottas et al. [39], heroin administration plays an enormously crucial role in predicting metabolite percentage distribution. As a consequence of molecular chemical properties, the 6-MAM concentration in brain tissues is much higher than the concentration in blood, whereas only a small amount of heroin is transferred through the blood–brain barrier [39]. Emphasizing the connection between forensic postmortem diagnostics and the 6-MAM molecule, a wide detection window seems to be useful [40]. Additionally, 6-MAM accumulation in vitreous humor and urine, where it is less likely to undergo enzymatic reactions, highlights the importance of this metabolite in determining long-term exposure. Furthermore, Thaulow et al. [41] indicated that the morphine/codeine ratio is a relevant feature of heroin intake even if 6-MAM remains below the limit of detection [41,42].

Codeine is metabolized mainly to one active substance, codeine-6-glucuronide, which represents almost 80% of codeine metabolites. Another metabolite is norcodeine, which is eventually transformed into normorphine [43] and shows analgesic properties, which is the reason for its usefulness as a codeine intoxication indicator [44]. Research conducted by Rees et al. [42] demonstrated that, although the results obtained from vitreous humor and blood samples are not fully correlated, access to both of them may have a significant role within the assessment.

The analgesic influence of morphine is calculated mostly on the basis of the activity of morphine-6-glucuronide, which is characterized by much higher affinity to opioid receptors than the parent substance and the second metabolite, morphine-3-glucuronide [45]. Recently, a thoroughly validated quantification method for morphine metabolites was reported by the Portuguese group of Oliveira et al. [46], and by testing various matrices, they obtained very encouraging sensitivity and accuracy results.

Cannabidiol (CBD) has recently gained more interest due to its multiple properties, including antioxidant, anti-inflammatory, and anticonvulsant effect. Citti et al. [47] conducted an analysis of rat brain tissue, exposing biochemical implications due to CBD intake. Moreover, they proved that untargeted metabolomics is a decent approach for examination of the effects in the brain tissues after an oral administration of a single, high dose of CBD. 

Another group of chemical substances that have attracted considerable interest is prescription drugs, such as sedative-hypnotic drugs, and the concentrations of these drugs can be measured with a high degree of certainty. As another example, Piotrowski et al. [48] developed a method that can be used with different types of samples to determine the level of zolpidem and its metabolite content. Notably, the evaluated method is suitable for both clinical and forensic cases and does not require large financial outlays.

To investigate the drug metabolite levels, the GC-TOF/MS technique has frequently been reported. According to results obtained by numerous research groups [49,50], the application of this particular analytical platform can lead to the determination of several chemical compounds as potential abuse biomarkers. Other methods examined include GC-MS and LC-MS with different mass analyzers, although these methods require excessive assays.

#### 3.2.2. Ethyl Alcohol and Ethanol Substitutes

According to a World Health Organization statistical report, the excessive consumption of ethyl alcohol (ethanol) is considered the cause of death for more than 3 million people worldwide each year. In many cases, determination of the exact cause of death is important for forensic and medical opinions, and the circumstances of death do not always allow a clear and correct assessment of the impact of ethyl alcohol. Only determination of the blood ethyl alcohol concentration at a sufficiently high level allows the conclusion that death was caused by acute alcohol intoxication.

Ethanol and its substitutes undergo various chemical reactions. Its consumption can be described by two main groups: products of oxidative and nonoxidative metabolic pathways. Extensive expertise on ethanol metabolites is incredibly crucial, as ethanol contributes to a wide range of diseases. Ethyl alcohol metabolites, which are formed simultaneously, are present in blood and urine and also accumulate in body tissues at high levels. Throughout the first phase, ethanol is metabolized by alcohol dehydrogenase (ADH) into acetaldehyde at a level of approximately 90%. Then, acetaldehyde is gradually transformed into acetate via the mitochondrial enzyme aldehyde dehydrogenase (ALDH2).

Although these processes constitute the major part of ethanol metabolism, some metabolites are the products of nonoxidative reactions. Notably, ethyl sulfate (EthS), ethyl glucuronide (EthG), phosphatidylethanol (PEth), and fatty acid ethyl esters have a more extensive window of detection and are associated with special properties that render them suitable as biomarkers. Moreover, nonoxidative metabolites seem to be more specific than other molecules present in, for example, the bloodstream or liver tissue [51,52]. This small percentage of all metabolites, which can be called alcohol intake markers, have a much longer detection period in human tissues and fluids and are, therefore, promising in alcohol abuse diagnosis [53]. Notably, these metabolites tend to accumulate in products of human skin cell differentiation, such as hair or nails [54,55], allowing analysts to perform extensive laboratory assays. Among the molecules, PEth is regarded as the best indicator of alcohol consumption, but published results may raise some doubts. On the one hand, PEth is defined by high stability when stored at −80 °C; on the other hand, assays carried out by Aradottir et al. [56] showed that PEth could be equally produced in vitro in the presence of ethanol, causing the final result of the measurement to not fully correspond to the actual concentration after death. Although PEth is detectable with satisfying and reliable sensitivity and selectivity, these aspects allow us to only suspect alcohol-related problems and do not reflect the exact amount of alcohol consumed [57].

However, a study on the L-kynurenine molecule (an indirect metabolite of ethanol) presented encouraging results, which may contribute to the wider usefulness of this parameter among forensic or clinical cases. Badawy et al. [58] verified the correlation between kynurenine levels and ethyl alcohol consumption by examining 10 healthy subjects after different dosages of ethanol. They demonstrated that ethanol elevated the blood concentration of kynurenine, which may further affect the central serotonergic system. Moreover, as animal experimental testing has shown, this scheme might help regulate the drinking pattern [59].

## 4. Biological Materials Commonly Used in Forensic Medicine

Forensic medicine focuses on the examination of exceedingly specific and various types of tissues obtained during forensic autopsy. As Álvarez-Sánchez et al. [60] mentioned, more than one possible biological material is available, allowing researchers to select the most suitable material. By reviewing the articles, most of the efforts were found to involve the use of plasma due to its availability, ease of sampling, and wide range of present metabolites. Nevertheless, currently, most metabolomics’ attempts have proceeded in antemortem, not postmortem, cases.

### 4.1. Types of Biological Materials in Forensic Samples

**Figure 1:**
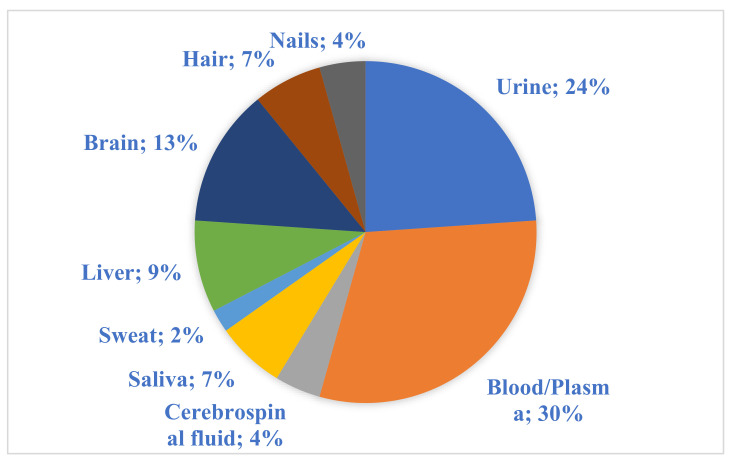
The percentage of each type of biological material used among the reviewed original papers.

The selection of a convenient sample type is a critical decision in each test. Selecting an appropriate method is essential to obtain satisfactory and validated results. The usefulness of each sample type has the strongest impact on selection. Due to extensive examination of biological matrices and a substantial source of information in previously performed assays, blood and urine samples are commonly selected [10] (Figure 1).

Some types of body fluids are constantly being used in forensic analysis due to their simplicity (the process of sample preparation is not very time consuming) and approachability while sampling. These features precisely describe the blood and urine samples mentioned above. With deep insight into catabolic and anabolic reactions occurring in the human body, metabolite levels can be efficiently determined, and an organism’s condition can be estimated [61].

#### 4.1.1. Peripheral Blood

Blood, with its fractions, has been the most frequently used biological material in metabolomics’ assays. Previously, as reported by Coe et al. [62], blood was suggested to carry most chemical substances in the body and was frequently used as an analytical sample because other specimens, such as vitreous humor, require more complex methodological procedures. Blood contains not only blood cells but also their metabolites, proteins, and damaged body cell content. The percentage of each fraction is determined by age, sex, and environmental factors; however, blood is a very valuable type of sample in forensic medicine because of its regularity between different species [10]. Certainly, blood composition and blood physicochemical properties vary between antemortem and postmortem cases. As a result of tissue breakdown processes, blood becomes up to 20 times more acidic than normal, which affects every further stage of the analysis, as a lower pH activates several enzymes that prevent blood clotting [63].

#### 4.1.2. Urine

Urine is generally collectable in relatively enormous quantities, not only for antemortem examinations but also after death. Sampling is noninvasive, and the material preparation process is described as being one of the simplest processes. Low-protein concentrations affect the utility of the sample preparation process. On the other hand, urine may contain more conjugated compounds, such as the products of drug metabolism reactions and the products of other catabolic reactions occurring in an organism. As mentioned by Álvarez-Sánchez et al. [60], urine samples, as well as blood samples, can provide information about the general health condition of a patient. However, the urine composition provides average or delayed information on recent internal alterations and environmental effects, whereas blood is considered a source of up-to-date data of an organism’s condition at the exact time of sampling. Nonetheless, volume correction is necessary when extracting urine samples because the molecular composition and concentrations strongly correspond to the sample volume [64,65]. Several assays for urine screening for drugs using metabolomics’ methods have been performed, and confirmation of the presence of up to 62 drugs with their metabolites was found in urine by the UHLPC-QTOF/MS method, which was a promising outset for a specific database. In addition, Tsai et al. [66] simplified the sample preparation method, which did not interfere with the high selectivity, sensitivity, and precision.

#### 4.1.3. Hair

Hair, which is considered a product of cell differentiation from human skin tissue, has always been of particular interest to scientists working on chemical testing to diagnose long-term drug use [67]. Strongly emphasized by Kintz et al. [68,69], the advantages of hair samples, which can have extended detection windows (correlated to hair length) and entail noninvasive sample collection, had a meaningful impact on enhancing efforts to broaden the scope of hair application. The significance of and interest in “-omics” (mostly genomics) applications in hair analysis have dramatically increased over the last few years as access to more specific and precise experimental methods has increased [70]. Heavy metal intoxication was investigated 50 years ago to verify drug abuse or chronic poisoning. However, the influence of environmental factors on the hair structure and on the level of incorporation by chemical substances externally and internally remains controversial. Genomics’ assays might provide meaningful evidence in sexual assault cases when alternative evidence materials are unavailable, as presented in the research conducted by Opel et al. [70]. On the basis of genetic analyses, they obtained a satisfactory concentration of DNA isolate despite the significant level of nucleic acid degradation. However, in some assays, the amount of nuclear DNA present might not be sufficient and may vary between particular cases due to personal features. Nevertheless, such DNA might constitute a highly supportive source of information. One of the obvious benefits of hair sample implementation is a longer detection window for some metabolites than that for blood or urine samples, which provides an opportunity to explore a patient’s pharmacological history [71,72]. Furthermore, a study of different hair sections may provide information about drug intake chronology given that hair grows approximately 1 cm per month. In addition, hair sampling is usually less problematic, and surviving victims are more eager to participate in clinical examinations and toxicological sampling, which seem to be crucial in forensic cases [73,74].

#### 4.1.4. Nails

Nail plates on both the fingers and toes are composed of a protein called alpha-keratin and are characterized by a strong and resistant structure [75]. Compared with other types of forensic samples, nails are defined as more advantageous and effective because of their resistance and capacity to accumulate a huge range of substances, such as drugs, toxins, and their metabolites. As demonstrated in the research performed by Cappelle et al. [76], nails have already been successfully used both in forensic analyses and clinical examinations, constituting a good supplementary matrix to blood or urine analysis results, as they can be correlated to obtain more reliable results.

The uniqueness of nails as forensic samples is related to a broad detection window and high resistance to environmental conditions. Nails as a source of expertise provide the opportunity to examine longer periods of chemical substance activity (as nails grow much more slowly than hair). Moreover, nails are favorable forensic analytical samples when the amount of hair is not adequate (i.e., during chemotherapy), and the collection process is less invasive, as the cut ends of the nail plate might constitute an analytical sample [10]. Importantly, in forensic assays, toenails appear to be more reliable than hand nails because the threat of contamination connected to handling drugs is limited. The lack of melanin in nails might influence metabolite concentration differences compared with other types of samples. As demonstrated in the past by Reid and Green et al. [77,78], the presence of melatonin enhances metabolite accumulation. Nail analysis has already been studied over the years and is used as a diagnostic tool for metal and drug intoxication [79]. As Alexiou et al. [80] verified, the metal concentration in children’s nail samples varied not only between the genders of the examined groups but also among ages, with a tendency to decrease after infancy when the values were highest. An issue that Palmeri et al. [59] highlighted is that drug concentrations in nails have been strongly correlated with actual intake. However, most studies focus on continuous exposure [81], but research regarding the detection of a single contact with the toxin is insufficient.

#### 4.1.5. Saliva

Saliva as an analytical sample has been defined as more compelling than other types of biological materials or body fluids. The process of sample collection and storage is economical and characterized by high sensitivity and a significant correlation with blood molecule concentrations [82]. The saliva composition remains relatively stable, especially as a dehydrated sample, and saliva might constitute a source of DNA, toxin metabolites, and other biomarkers, such as cortisol or alpha-amylase. These data can be potentially useful not only for drug level monitoring but also as a screening tool for multiple diseases (autoimmunological or cardiovascular) [83]. A meaningful aspect of saliva samples is susceptibility to environmental, physiological, and pathological factors. The most prevalent determinants to differentiate are annual biochemical changes, nutrition intake and the quality of nutrients, the organism’s condition, and drug intake [84]. Regarding composition alterations in saliva, Larsen et al. [85] asserted that the results obtained from the same patient’s sample differ significantly in a period of not only months but also hours. Comprehensive metabolite profiling of saliva has already been performed [86], and the results have been demonstrated to be useful in disease diagnosis and are already readily used, especially in dental disorder diagnosis [84] or biomarker discovery. More importantly, Zhang et al. [87] demonstrated that saliva sampling is a relatively noninvasive and harmless method that should reduce patients’ fear and increase their likelihood of participating in regular and repetitive examinations. In addition, saliva can provide detailed information describing and characterizing microbiome changes during the decomposition process.

#### 4.1.6. Sweat

The popularity of sweat samples is attracting more attention, as sampling has become one of the least invasive among most samples regularly used in forensic analysis. Researchers have focused on sweat usefulness in the clinical field, mostly focusing on biomarkers of lung cancer and other respiratory tract diseases. The sweat in the routine diagnostic protocol for cystic fibrosis has been successfully analyzed for many years and is constantly considered the gold standard despite the availability of more advanced and modern tests [88]. Many attempts using both liquid chromatography-tandem mass spectrometry (LC-QTOF/MS) and gas chromatography mass spectrometry (GC-TOF/MS) have been carried out and might have encouraged scientists to emphasize the value and efficacy of sweat samples in the context of biomarker discovery. For instance, with the aid of the LC-QTOF/MS method, Calderon et al. [89] collected extensive data specific to sweat components.

#### 4.1.7. Internal Organ Tissue

Not only connective tissues or excrements can be considered valuable sample sources. In forensic analyses, the easy sampling procedure is not the priority, and specimens can be obtained from random body organs. As we consider an organism to be a complete entity, organs contain particular quantities of various molecules present in, i.e., whole-blood or urine samples.

##### Liver

Studies on liver tissue have shown that this type of material can provide us with valuable information about individual medical treatment and diseases. Similar to the kidneys, liver tissue is also exposed to the harmful impact of substances supplied to the organism throughout the whole lifespan. The presence of certain metabolites may indicate serious liver failure caused by overdosed stimulants and medications [90,91], and the results might help determine the medical history of the patient. Furthermore, postmortem liver samples have shown fewer metabolomics’ alterations than antemortem tissue samples. The study performed by Lin et al. [92] advocates the liver as a valuable source of data when using NMR spectroscopy. Carefully selected analytical methods should minimize the probability of interference and increase the probability of finding metabolomic similarities between samples.

Notably, in the experiment conducted by Harada et al. [93], the authors examined fasting men’s plasma samples to verify the influence of alcohol intake on the blood metabolome and explored potential biomarkers of alcohol-dependent liver injury. They were able to successfully identify a group of molecules that are likely to be associated with excessive daily ethanol consumption. Another group of researchers, Griffin et al. [94], tried to find an appropriate animal model to best reflect the early stage metabolic changes in fatty liver disease in humans. They claimed that meaningful variations exist between populations that impact fatty liver disease occurrence; the differences between apparent baseline metabolism in liver tissue and the genetic background seem to be largely responsible.

##### Brain

Most recent studies have focused on examining biofluids such as plasma or urine, as they provide wider insight into all metabolic processes, and brain tissue accessibility is limited to samples of animal origin or postmortem human brain studies. Consequently, at the moment of death, remaining amounts of metabolites are destroyed because of the brain’s oxygen demand [95]. Notably, one of the crucial discrepancies of the brain over the rest of the organs is blood–brain barrier isolation. As a consequence, only several types of molecules are capable of penetrating this highly secured part of the human body. Due to the complexity of brain tissue, extracting and determining all of the metabolites and lipids of interest are nearly impossible. These chemical substances are characterized by diverse properties, and their final concentrations in a sample are affected by the choice of solvents, their ratios, etc. As presented by Naz et al. [96], various conditions influence the selection of the final method for brain tissue analysis. Moreover, the strategies and procedures associated with brain tissue analysis are burdened with far-reaching intricacies such as brain heterogeneity or sample storage, given the thermal lability of some molecules [97]. Recently, several articles have been published on the potential application of different analytical methods and the usefulness of brain tissue in regard to metabolite distributions in different parts of brain regions. Studies have mostly focused on mental [98] and neurodegenerative disorders [99] such as Alzheimer disease. Discussing the issue of neurotransmitters’ metabolome among patients suffering from alcohol addiction, Kashem et al. [98] demonstrated significantly important differences between neurotransmitters’ concentration in different alcoholic individuals’ brain regions, emphasizing the content of gamma-aminobutyric acid (GABA). With satisfactory specificity and accuracy, researchers were able to identify molecules that might later be determined to be prognostic biomarkers. Another assay performed by Thierauf-Emberger et al. [100] compared the presence of ethanol molecules in two types of samples—blood and brain tissue—and emphasized that although the brain is the target organ of ethanol, brain concentrations are usually lower.

##### Kidney

The kidney is responsible for blood filtration and urine production, implying that it has constant contact with all of the metabolites produced in the human body. Renal tissue undergoes many meaningful changes after death. The metabolomics’ assay performed by Mora-Ortiz et al. [101] showed meaningful differences between the concentrations of amino acids in kidney samples before and after death and reported that the kidney shows the most changes during the postmortem period, as verified over three time points. Forty-three different metabolites were demonstrated to be associated with postmortem fluctuations occurring after death.

### 4.2. Importance of Sample Preparation

Focusing on the main objective of the study, the complexity of the sample preparation process could differ. More detailed results are expected, and the sample preparation procedure is usually more complicated and long [10]. Depending on the research goal—whether an untargeted or a targeted metabolomics’ approach is involved—researchers need to focus on a group of molecules or on only some particular molecules. When searching for the most effective results, both the analytes and matrix nature must be studied. Depending on the type and characteristics of analyzed biological material, the most appropriate methods of homogenization and compound extraction must be selected because the final analysis result depends on correct implementation of these stages [97]. Another important aspect is the laboratory limitations and the uneven conditions between them that may lead to several protocol differences and incorrect interpretation of the results. As research focuses on small molecules that undergo many chemical reactions and transformations in the body, minimizing analysis differences is extremely important, as smaller differences correspond to more reliable results.

## 5. Methods Used in Forensic Instrumental Analysis

The complexity of analytical instrumentation used within forensic medicine has enormously changed and improved over the last decades. A simple change in reagent color is no longer sufficient to support an indisputable claim. As an imminent result, a wide range of methods are currently accessible in thanatometabolomics, and mass spectrometry (MS) and nuclear magnetic resonance spectroscopy (NMR) seem to be the most popular [102,103]. However, in recent years, other modern electro- and liquid-phase separation techniques have gained popularity due to their reliability and practical aspects [104]. Striving for the most accurate results, these techniques are often combined with mass spectrometry. However, the negative consequences of partial automation and the high cost and time requirements of targeted profiling cannot be overlooked and may be the reason why untargeted analyses are still more frequently conducted and reported. Depending on the purpose of the experiment, different analytical techniques are applied. NMR spectroscopy has been reported to be used in metabolomics since the very beginning and is characterized by several features that support its application. This method can be used both for quantitative and qualitative assays, as demonstrated by highly reproducible results and nondemanding sample preparation procedures [105]. Hence, combining NMR spectroscopy results with UPLC-QTOF-MS may constitute a valuable and indisputable supplement to the results obtained. Zhang et al. [106] combined the two methods mentioned to fully analyze the molecules of interest, as some of them were undetectable using one method but visible while applying another. Through this attempt, the authors emphasized the necessity of complex multiplatform analysis, which allows the acquisition of a more extensive metabolite panel with the application of only one technique. Notably, however, when using ^1^H NMR, solvents and other chemical contaminants may generate visible signals and interfere with the signal of the substance of interest [107]. Furthermore, NMR analysis does not require sample extraction and is considered a nondestructive technique [97]. However, these features may result in low sensitivity as a limitation and can preclude the detection of compounds at low concentrations. This phenomenon is increasingly desirable, especially because everyone is concentrating on environmental contamination problems and struggling to minimize the use of chemical compounds in laboratories. What could be a significant limitation while using ^1^H NMR is the availability of well-collected research material, sampled in a sufficient volume/quantity. In addition, another disadvantage is the availability of NMR equipment, which is not so widespread due to the huge price of the instruments [16].

Meanwhile, mass spectrometry analysis is important among nontargeted metabolomics’ approaches. Its exceptional availability and dissemination in research centers, combined with a short analysis time and high selectivity, reproducibility, and sensitivity, increase the method’s popularity. Mass spectrometry is also the only method where the results for brain or liver tissue are the least likely to involve concern regarding interpretation, as recent analyses have demonstrated [93,108,109].

In the example of mouse liver tissue, Kang et al. [109] carried out an experiment demonstrating the utility of the UPLC-QTOF/MS-based metabolomics’ approach in forensic science, as an assumption had been proposed concerning a few possible candidates for PMI biomarkers. Furthermore, Isbell et al. [110] conducted an analysis verifying the total morphine concentration using both CE-ESI-QTOF/MS and HPLC-MS methods at the 99% confidence level and declared no statistically significant difference between the results. Another group of researchers, Nielsen et al. [111], analyzed blood samples from intoxicated car drivers using UPLC-HR-TOF/MS and demonstrated that relevant results could be obtained from retrospective screening data for metabolic profiling in relation to drug metabolism and physiological and toxicological effects.

**Figure 2:**
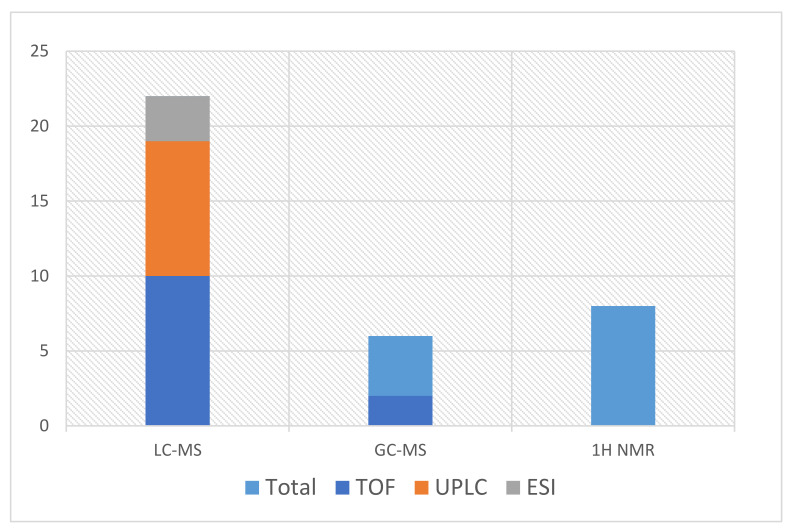
The use frequency of different analytical methods.

Furthermore, mass spectrometry coupled with gas chromatography (GC) or liquid chromatography (LC) allows the analyst to find a reasonable solution for problems that are caused by the complexity of the sample [82]. By combining GC-MS with LC-MS, the main conclusion is that GC is appropriate only for volatile compounds, whereas liquid chromatography covers a wider range of chemical compounds without the need for derivatization. As Maenhout et al. [51] admitted, this quality is crucial because it allows multiple types of samples to be analyzed, including those with a complex matrix. The popularity of each mentioned analytical method is presented in the scheme in Figure 2.

As the needs of scientists are evolving, other methods have been developed and have recently been commonly used in laboratory practice. One can list normal phase (NP), reversed-phase (RP) and hydrophilic interaction chromatography (HILIC) [96], direct analysis in real time (DART), desorption electrospray ionization mass spectrometry (D-ESI/MS) [112], or matrix-assisted laser desorption/ionization (MALDI), which rely on oxidation processes and are especially used for polymers, proteins, and nucleic acids [112]. The difficulty that commonly occurs is interference with a complex matrix during the analysis of small molecules [113]. Therefore, while preparing an assay consisting of biological samples, the exact composition of the matrix is hardly unknown, which creates other problems affecting the reliability of the results. Additionally, secondary-ion mass spectrometry (SIMS) [114] and laser ablation electrospray ionization tandem mass spectrometry (LA-ESI-MS/MS) have an immense role in observing reliable concentrations of drugs and their metabolites without very rough and time-consuming derivatization or extraction processes [115].

## 6. Method Development

Contributing to the development of less time-consuming metabolomics’ methods, the main trend is to obtain satisfactory results within a shorter period with lower laboratory outlays. Samples can be collected from different body organs and fluids, which is judgmentally appropriate and promising in forensic medicine. Comparing and correlating the results for samples from various sources (e.g., brain tissue, plasma, and vitreous humor) collected from one person to correctly determine different metabolic disturbances may also facilitate the establishment of the cause of death. Moreover, combining two or more techniques will positively influence the reliability of the presented results. Additional methodological aspects include economic and financial criteria in experimental analyses. With faster overall analytical processes and more automatized equipment, the metabolomics’ approach in forensic science will soon become increasingly popular. An overview of the most commonly used analytical methods and types of samples is presented in Table 1.

## 7. Conclusions

Although various types of analytical assays have been implemented over the last decades in response to the tremendous requirements of forensic science, the ongoing need for increasingly refined methods is goading the scientific community to seek alternative solutions. Forthcoming research involves a better focus on postmortem sample specificity and exceptional methods for sample preparation to achieve the most accurate and realistic results using modern separation techniques. Most importantly, the primary concern in analytics should be the selection of the most effective extraction method, as tissue extraction is one of the most limiting steps in any metabolomics’ study.

## Figures and Tables

**Table 1:** Overview of the analytical methods and types of samples used.

Reference	Method	Type of Sample	Sample Preparation	Analitycal Problem	Analytical/Validation Conditions
Griffin et al. [94]	^1^H NMR	Liver	Frozen tissue stored at −80 °C, homogenization	Steatosis caused by orotic acid	600.2 MHz Lvl. of significacnce = 0.005
Holmes et al. [27]		Urine	Samples buffered and internally calibrated	Detection of drug metabolome	600.29 MHz in flow-injection mode
Lin et al. [92]		Muscle	Tissue homogenization and extraction	Evaluation of different extraction methods/Tissue metabolome	500.11 MHz
Maher et al. [61]		Urine	-	The effect of long-term storage conditions on human urine metabolome	600 MHz
Mora-Ortiz et al. [101]		Heart, kidney, liver, spleen	Freezing and homogenization	Identification of metabolic biomarkers of the time of death	700 MHz
Welije et al. [102]		Urine	Simplified synthetic urine	The use of targeted profiling for mixture analysis	600 MHz
Huo et al. [99]	UPLC-MS/MS	Brain, plasma	Plasma sample derivatized with phenyl isothiocyanate Homogenized brain tissue sample	Determination of acylcarnitines, sphingolipids, glycerophospholipids	Biocrates AbsoluteIDQ^®^ p180 Kit
Gottas et al. [39]		Brain, blood	Brain tissue homogenate and blood samples mixed with reagents	Determination of heroin levels	LOD = 0.07 ng/mL for heroine, 0.26 ng/mL for morphine, 0.36 ng/mL for 6-MAM, 0.23 ng/mL for M3G; LLOQ = 3 ng/mL for heroine, 1.7 ng/mL for morphine, 2 ng/mL for 6-MAM, 2.2 ng/mL for M3G
Kang et al. [109]	UPLC-QTOF-MS	Liver	Homogenization and extraction	Metabolite changes related to postmortem interval	Column: ACQUITY UPLC column (BEH C18) 2.1 × 100 Column, (Waters, Milford, MA, USA)
Tsai et al. [66]		Urine	Urine diluted with water and centrifugated	Screening and confirmation of 62 drugs of abuse and their metabolites	LOD = 2.8 and 187.5 ng/mL for 62 metabolites
Tuhalow et al. [41]	UHPLC-MS/MS	Blood, urine, vitreous humour	-	Postmortem heroin levels’ determination	LOQ = 0.0033 mg/L and 0.0086 mg/L for 6-AM and morphine, 0.0090 mg/L for codeine, and 0.014 mg/L for M3G and M6G in blood, pericardial fluid, and vitreous humor
Harada et al. [93]	CE-MS	Plasma	Centrifugation and extraction	Novel biomarkers of alcohol intake	-
Gimenez-Gomez et al. [59]	HPLC, GC-MS	Plasma, brain	Samples’ deproteinization and homogenization (brain tissue)	Reduction of EtOH consumption induced by KYN and KYNA increments	Column: HR-80; 80 mm × 4.6 mm, 3 µm
Aradottir et al. [56]	HPLC	Liver, lung, spleen, heart, muscle, blood	Tissue homogenization and extration; blood extraction	Postmortem concentration of PEth in blood and organs influenced by storage conditions	Column: Licrosphere 100 DIOL, 5-m particle size
Gonzalez Riano et al. [108]	LC-MS	Brain	Tissue homogenization and extraction	Postmortem changes in hippocampus	-
Myint et al. [11]		Cereprospinal fluid	Protein-free samples were passed through an Oasis MCX 96-well plate cartridge; etuates were evaporated	Cationic metabolome analysis	LOD = 0.3–9.9 pmol
Andersen et al. [38]	LC-MS/MS	Plasma	Organic phase evaporation	Importance of heroin and its metabolites in eliciting a behavioral response in mice	LOD = 0.0065 mg/L for M3G, 0.00060 mg/L for M6G, 0.00049 mg/L for morphine, 0.00033 mg/L for 6MAM, and 0.00096 mg/L for heroin
Leon et al. [65]		Urine, semen	Addition of β-glucuronidase, incubation, SPE procedure	HMB determination	LOD = 0.027 and 0.103 ng/mL (urine) LOD = 1–3 ng/mL (semen)
Kashem et al. [98]		Brain	Tissue homogenization	Neurotransmitter metabolome and protein expression changes of humans exposed to heavy, long-term ethanol consumption	Column: BEH C18 (150 mm 9 2.1 mm; Waters), with 1.7-m particle size
Kintz et al. [71]		Blood, urine	-	Alcohol-related EtG and FAEE presence	-
Kintz et al. [69]		Hair	Decontamination, pulverization, incubation, and extraction of the sample	Hair analysis in postmortem toxicology	-
Shima et al. [73]		Hair	Sample decontamination, pulverization, and extraction	Zolpidem incorporated into hair	LOD = 50 fg/2-cm hair Recovery = 48% Precision RSD = 2.7% Intraday accuracy = 3.8%
Alvarez-Sanchez et al. [64]	LC-ESI-MS/MS	Urine	Enzymatic hydrolysis followed by mini-SPE procedure	Determination of free and glucuronide-conjugated female steroid hormones	Column: Agilent Zorbax Eclipse XDB-C18 (4.6 mm × 150 mm, 5 m particle size) LOD = 1.8–18 pg LOQ = 6–61 pg
Zheng et al. [57]		Blood	Lipid extracts of whole blood samples	Alcohol biomarker PEth in blood	R^2^= 0.994 LOD = 0.01 μmol/L
Zheng et al. [50]	GC-MS	Serum, urine	Serum and urine samples were pretreated, extracted, and derivatised	Identification of potential biomarkers of heroin abuse	Column: 10 m × 0.18 mm i.d. fused-silica capillary column chemically bonded with a 0.18-m DB5-MS stationary phase
Crunelle et al. [54]		Hair	Samples were mechanically pulverized	Correlations and gender influence on ethyl glucuronide concentration	LOQ = 2.10 pg/mg LOD = 0.70 pg/mg
Cappelle et al. [55]	GC-MS/MS	Nails	Sample decontamination, pulverization, and derivatization	Determination of EtG	Linearity range: 2–100 pg/mg LLOQ = 2 pg/mg
Stefanuto et al. [14]	GC-TOF-MS	VOC	Thermal desorption	VOC profile of human remains during early stages of decomposition	Columns: Restek Rxi-5Sil (5% phenyl–95% dimethyl polysiloxane) (Bellefonte, PA, USA) (30 m × 0.25 mm id × 0.25-μm df); Restek Rxi-17 (50% phenyl–50% dimethyl polysiloxane) (Bellefonte, PA, USA) (1.0 m × 0.15 mm id × 0.15-μm df)
Shima et al. [49]		Urine, plasma	Serum and urine samples were pretreated, extracted, and derivatised	Methamphetamine-induced acute intoxication influence on metabolome	Column: CP-SIL 8 (30 m × 0.25 mm i.d., 0.25-m film thickness, GL sciences)
Alvarez-Sanchez et al. [86]	LC-TOF/MS	Saliva	Hydrolysis (both basic and acidic) of saliva + ultrasound energy	Metabolomic profiling of human saliva	Column: Zorbax Eclipse XDB-C18 column (4.6 mm × 150 mm, 5-m particle size) Flow: 1 mL/min
Calderon-Santiago et al. [89]	LC-QTOF-MS/MS	Sweat	(a) Sweat hydrolysis under acid or alkaline conditions (b) Sweat cleanup and preconcentration by solid-phase extraction using C18 and hydrophilic centrifugal Micro SpinColumnTM systems	Method development for analysis of human sweat	Column: C18 reverse-phase (Mediterranean, 50 mm × 0.46 mm i.d., 3 m particle size) Flow: 0.8 mL/min
					Column: Luna hydrophilic interaction chromatography column (HILIC) (100 mm × 0.46 mm i.d., 3-m particle size) Flow: 0.6 mL/min
Krumbiegel et al. [81]		Nails	Ground by a ball mill and extracted twice	Usefulness of nail samples instead of hair for a general unknown screening (GUS) fordrugs	Column: Poroshell 120 EC-C18 column (2.1 × 100 mm, 2.7 µm, Agilent Technologies, Santa Clara, CA, USA)

## Data Availability

Data is contained within the review.

## References

[1] Kosmides A.K., Kamisoglu K., Calvano S.E., Corbett S.A., Androulakis I.P. (2013). Metabolomic Fingerprinting: Challenges and Opportunities. Crit. Rev. Biomed. Eng..

[2] Akçan R., Taştekin B., Yildirim M., Aydogan H.C., Sağlam N. (2020). Omics era in forensic medicine: Towards a new age. Turk. J. Med. Sci..

[3] Szeremeta M., Pietrowska K., Niemcunowicz-Janica A., Kretowski A., Ciborowski M. (2021). Applications of Metabolomics in Forensic Toxicology and Forensic Medicine. Int. J. Mol. Sci..

[4] Dinis-Oliveira R.J. (2018). Metabolism and metabolomics of opiates: A long way of forensic implications to unravel. J. Forensic Leg. Med..

[5] Mamas M., Dunn W., Neyses L., Goodacre R. (2010). The role of metabolites and metabolomics in clinically applicable biomarkers of disease. Arch. Toxicol..

[6] Griffiths W.J., Koal T., Wang Y., Kohl M., Enot D.P., Deigner H.-P. (2010). Targeted Metabolomics for Biomarker Discovery. Angew. Chem. Int. Ed..

[7] Nicholson J.K., Wilson I.D. (2003). Understanding ‘Global’ Systems Biology: Metabonomics and the Continuum of Metabolism. Nat. Rev. Drug Discov..

[8] Harrigan G.G., Maguire G., Boros L. (2008). Metabolomics in alcohol research and drug development. Alcohol Res. Health.

[9] Fiehn O., Town C. (2002). Metabolomics—The link between genotypes and phenotypes. Functional Genomics.

[10] Castillo-Peinado L., de Castro M.L. (2016). Present and foreseeable future of metabolomics in forensic analysis. Anal. Chim. Acta.

[11] Myint K.T., Aoshima K., Tanaka S., Nakamura T., Oda Y. (2009). Quantitative Profiling of Polar Cationic Metabolites in Human Cerebrospinal Fluid by Reversed-Phase Nanoliquid Chromatography/Mass Spectrometry. Anal. Chem..

[12] Theodoridis G., Gika H.G., Wilson I.D. (2008). LC-MS-based methodology for global metabolite profiling in metabonomics/metabolomics. TrAC Trends Anal. Chem..

[13] Gika H.G., Theodoridis G.A., Wilson I.D. (2008). Liquid chromatography and ultra-performance liquid chromatography–mass spectrometry fingerprinting of human urine: Sample stability under different handling and storage conditions for metabonomics studies. J. Chromatogr. A.

[14] Stefanuto P.-H., Perrault K., Stadler S., Pesesse R., Leblanc H.N., Forbes S.L., Focant J.-F. (2015). GC × GC–TOFMS and supervised multivariate approaches to study human cadaveric decomposition olfactive signatures. Anal. Bioanal. Chem..

[15] Meyer M.R., Maurer H.H. (2014). Toxicokinetics and Toxicogenetics. Handbook of Forensic Medicine.

[16] Locci E., Bazzano G., Chighine A., Locco F., Ferraro E., Demontis R., D’Aloja E. (2020). Forensic NMR metabolomics: One more arrow in the quiver. Metabolomics.

[17] Macdonald K., Krishnan A., Cervenka E., Hu G., Guadagno E., Trakadis Y. (2019). Biomarkers for major depressive and bipolar disorders using metabolomics: A systematic review. Am. J. Med. Genet. Part B Neuropsychiatr. Genet..

[18] Pang H., Jia W., Hu Z. (2019). Emerging Applications of Metabolomics in Clinical Pharmacology. Clin. Pharmacol. Ther..

[19] Locci E., Stocchero M., Noto A., Chighine A., Natali L., Napoli P.E., Caria R., De-Giorgio F., Nioi M., D’Aloja E. (2019). A 1H NMR metabolomic approach for the estimation of the time since death using aqueous humour: An animal model. Metabolomics.

[20] Vinayavekhin N., Saghatelian A. (2010). Untargeted Metabolomics. Curr. Protoc. Mol. Biol..

[21] Chighine A., Locci E., Nioi M., D’Aloja E. (2021). Looking for Post-Mortem Metabolomic Standardization: Waiting for Godot—The Importance of Post-Mortem Interval in Forensic Metabolomics. Chem. Res. Toxicol..

[22] DiMaio V., DiMaio D. (2001). Forensic Pathology. https://content.taylorfrancis.com/books/download?dac=C2009-0-01761-8&isbn=9781420042412&format=googlePreviewPdf.

[23] Kaliszan M., Hauser R., Kernbach-Wighton G. (2009). Estimation of the time of death based on the assessment of post mortem processes with emphasis on body cooling. Leg. Med..

[24] Henssge C., Madea B. (2007). Estimation of the time since death. Forensic Sci. Int..

[25] Buchan M., Anderson G. (2001). Time Since Death: A Review of the Current Status of Methods used in the Later Postmortem Interval. Can. Soc. Forensic Sci. J..

[26] Kugelberg F.C., Jones A.W. (2007). Interpreting results of ethanol analysis in postmortem specimens: A review of the literature. Forensic Sci. Int..

[27] Holmes E., Loo R.L., Cloarec O., Coen M., Tang H., Maibaum E., Bruce S., Chan Q., Elliott P., Stamler J. (2007). Detection of Urinary Drug Metabolite (Xenometabolome) Signatures in Molecular Epidemiology Studies via Statistical Total Correlation (NMR) Spectroscopy. Anal. Chem..

[28] Strimbu K., Tavel J.A. (2010). What are biomarkers?. Curr. Opin. HIV AIDS.

[29] Pesko B.K., Weidt S., McLaughlin M., Wescott D.J., Torrance H., Burgess K., Burchmore R. (2020). Postmortomics: The Potential of Untargeted Metabolomics to Highlight Markers for Time Since Death. OMICS.

[30] Jawor P., Ząbek A., Wojtowicz W., Król D., Stefaniak T., Młynarz P. (2019). Metabolomic studies as a tool for determining the post-mortem interval (PMI) in stillborn calves. BMC Vet. Res..

[31] Dai X., Fan F., Ye Y., Lu X., Chen F., Wu Z., Liao L. (2019). An experimental study on investigating the postmortem interval in dichlorvos poisoned rats by GC/MS-based metabolomics. Leg. Med..

[32] Aronson J.K. (2005). Biomarkers and surrogate endpoints. Br. J. Clin. Pharmacol..

[33] Steuer A.E., Brockbals L., Kraemer T. (2019). Metabolomic Strategies in Biomarker Research–New Approach for Indirect Identification of Drug Consumption and Sample Manipulation in Clinical and Forensic Toxicology?. Front. Chem..

[34] Steuer A.E., Raeber J., Steuer C., Boxler M.I., Dornbierer D.A., Bosch O.G., Quednow B.B., Seifritz E., Kraemer T. (2018). Identification of new urinary gamma-hydroxybutyric acid markers applying untargeted metabolomics analysis following placebo-controlled administration to humans. Drug Test. Anal..

[35] Dinis-Oliveira R.J., Carvalho F., Moreira R.F., Duarte J.A., Proença J., Santos A., Magalhães T. (2012). Clinical and Forensic Signs Related to Opioids Abuse. Curr. Drug Abus. Rev..

[36] Inturrisi C., Schultz M., Shin S., Umans J., Angel L., Simon E. (1983). Evidence from opiate binding studies that heroin acts through its metabolites. Life Sci..

[37] Way E.L., Kemp J.W., Young J.M., Grassetti D.R. (1960). The pharmacologic effects of heroin in relationship to its rate of biotransformation. J. Pharmacol. Exp. Ther..

[38] Andersen J.M., Ripel Å., Boix F., Normann P.T., Mørland J. (2009). Increased Locomotor Activity Induced by Heroin in Mice: Pharmacokinetic Demonstration of Heroin Acting as a Prodrug for the Mediator 6-Monoacetylmorphine in Vivo. J. Pharmacol. Exp. Ther..

[39] Gottas A., Øiestad E.L., Boix F., Vindenes V., Ripel A., Thaulow C.H., Mørland J. (2013). Levels of heroin and its metabolites in blood and brain extracellular fluid after i.v. heroin administration to freely moving rats. Br. J. Pharmacol..

[40] Dinis-Oliveira R.J., Vieira D.N., Magalhães T. (2016). Guidelines for Collection of Biological Samples for Clinical and Forensic Toxicological Analysis. Forensic Sci. Res..

[41] Thaulow C.H., Øiestad Å.M.L., Rogde S., Andersen J.M., Høiseth G., Handal M., Mørland J., Vindenes V. (2018). Can measurements of heroin metabolites in post-mortem matrices other than peripheral blood indicate if death was rapid or delayed?. Forensic Sci. Int..

[42] Rees K.A., Pounder D.J., Osselton M.D. (2013). Distribution of opiates in femoral blood and vitreous humour in heroin/morphine-related deaths. Forensic Sci. Int..

[43] Srinivasan V., Wielbo D., Tebbett I. (1997). Analgesic effects of codeine-6-glucuronide after intravenous administration. Eur. J. Pain.

[44] Haavik P.E. (2000). Kodein er prodrug—Virkestoffet er morfin. Tidsskr. Den Nor. Laegeforening.

[45] Paul D., Standifer K.M., Inturrisi C.E., Pasternak G.W. (1989). Pharmacological characterization of morphine-6 beta-glucuronide, a very potent morphine metabolite. J. Pharmacol. Exp. Ther..

[46] Oliveira A., Carvalho F., de Pinho P.G., Remião F., Medeiros R., Dinis-Oliveira R.J. (2014). Quantification of morphine and its major metabolites M3G and M6G in antemortem and postmortem samples. Biomed. Chromatogr..

[47] Citti C., Palazzoli F., Licata M., Vilella A., Leo G., Zoli M., Vandelli M.A., Forni F., Pacchetti B., Cannazza G. (2018). Untargeted rat brain metabolomics after oral administration of a single high dose of cannabidiol. J. Pharm. Biomed. Anal..

[48] Piotrowski P., Bocian S., Śliwka K., Buszewski B. (2015). Simultaneous analysis of zolpidem and its metabolite in whole blood and oral fluid samples by SPE-LC/MS for clinical and forensic purposes. Adv. Med. Sci..

[49] Shima N., Miyawaki I., Bando K., Horie H., Zaitsu K., Katagi M., Bamba T., Tsuchihashi H., Fukusaki E. (2011). Influences of methamphetamine-induced acute intoxication on urinary and plasma metabolic profiles in the rat. Toxicology.

[50] Zheng T., Liu L., Aa J., Wang G., Cao B., Li M., Shi J., Wang X., Zhao C., Gu R. (2013). Metabolic phenotype of rats exposed to heroin and potential markers of heroin abuse. Drug Alcohol Depend..

[51] Maenhout T.M., De Buyzere M.L., Delanghe J.R. (2013). Non-oxidative ethanol metabolites as a measure of alcohol intake. Clin. Chim. Acta.

[52] Zakhari S. (2006). Overview: How Is Alcohol Metabolized by the Body?. Alcohol Res. Health.

[53] Dinis-Oliveira R.J., Magalhães T., Moreira R.F., Proença J.B., Pontes H., Santos A., Duarte J.A., Carvalho F. (2014). Clinical and forensic signs related to ethanol abuse: A mechanistic approach. Toxicol. Mech. Methods.

[54] Crunelle C.L., Cappelle D., Flamand E., Cox J., Covaci A., De Doncker M., Van Nuijs A.L.N., Michielsen P., Yegles M., Neels H. (2016). Ethyl glucuronide in hair of non-excessive alcohol consumers: Correlations and gender influence. Forensic Toxicol..

[55] Cappelle D., Neels H., Yegles M., Fransen E., Dueffels K., Bremenfeld S., Maudens K.E., Van Nuijs A.L.N., Covaci A., Crunelle C.L. (2016). Ethyl glucuronide in nails: Method validation, influence of decontamination and pulverization, and particle size evaluation. Forensic Toxicol..

[56] Aradottir S., Seidl S., Wurst F.M., Jönsson B., Alling C. (2004). Phosphatidylethanol in Human Organs and Blood: A Study on Autopsy Material and Influences by Storage Conditions. Alcohol. Clin. Exp. Res..

[57] Zheng Y., Beck O., Helander A. (2011). Method development for routine liquid chromatography–mass spectrometry measurement of the alcohol biomarker phosphatidylethanol (PEth) in blood. Clin. Chim. Acta.

[58] Badawy A.A.-B., Doughrty D.M., Marsh-Richard D.M., Steptoe A. (2009). Activation of Liver Tryptophan Pyrrolase Mediates the Decrease in Tryptophan Availability to the Brain after Acute Alcohol Consumption by Normal Subjects. Alcohol Alcohol..

[59] Giménez-Gómez P., Pérez-Hernández M., López M.D.G., Vidal R., Abuin-Martínez C., O’Shea E., Colado M.I. (2018). Increasing kynurenine brain levels reduces ethanol consumption in mice by inhibiting dopamine release in nucleus accumbens. Neuropharmacology.

[60] Álvarez-Sánchez B., Priego-Capote F., de Castro M.D.L. (2010). Metabolomics analysis I. Selection of biological samples and practical aspects preceding sample preparation. TrAC Trends Anal. Chem..

[61] Maher A.D., Zirah S.F.M., Holmes A.E., Nicholson J.K. (2007). Experimental and Analytical Variation in Human Urine in 1H NMR Spectroscopy-Based Metabolic Phenotyping Studies. Anal. Chem..

[62] Coe J.I. (1993). Postmortem Chemistry Update Emphasis on Forensic Application. Am. J. Forensic Med. Pathol..

[63] Donaldson A.E., Lamont I.L. (2014). Estimation of post-mortem interval using biochemical markers. Aust. J. Forensic Sci..

[64] Álvarez Sánchez B., Priego-Capote F., Jiménez J.R., Luque de Castro M.D. (2008). Automated solid-phase extraction for concentration and clean-up of female steroid hormones prior to liquid chromatography–electrospray ionization–tandem mass spectrometry: An approach to lipidomics. J. Chromatogr. A.

[65] León Z., Chisvert A., Tarazona I., Salvador A. (2010). Solid-phase extraction liquid chromatography–tandem mass spectrometry analytical method for the determination of 2-hydroxy-4-methoxybenzophenone and its metabolites in both human urine and semen. Anal. Bioanal. Chem..

[66] Tsai I.-L., Weng T.-I., Tseng Y.J., Tan H.K.-L., Sun H.-J., Kuo C.-H. (2013). Screening and Confirmation of 62 Drugs of Abuse and Metabolites in Urine by Ultra-High-Performance Liquid Chromatography-Quadrupole Time-of-Flight Mass Spectrometry. J. Anal. Toxicol..

[67] Tridico S. (2014). Hair: Animal. Wiley Encyclopedia of Forensic Science.

[68] Kintz P., Villain M., Cirimele V. (2006). Hair Analysis for Drug Detection. Ther. Drug Monit..

[69] Kintz P. (2004). Value of hair analysis in postmortem toxicology. Forensic Sci. Int..

[70] Opel K.L., Fleishaker E.L., Nicklas J.A., Buel E., Mccord B.R. (2008). Evaluation and Quantification of Nuclear DNA from Human Telogen Hairs. J. Forensic Sci..

[71] Kintz P., Nicholson D. (2013). Interpretation of a highly positive ethyl glucuronide result together with negative fatty acid ethyl esters result in hair and negative blood results. Forensic Toxicol..

[72] Namera A., Urabe S., Saito T., Torikoshi-Hatano A., Shiraishi H., Arima Y., Nagao M. (2013). A fatal case of 3,4-methylenedioxypyrovalerone poisoning: Coexistence of α-pyrrolidinobutiophenone and α-pyrrolidinovalerophenone in blood and/or hair. Forensic Toxicol..

[73] Shima N., Sasaki K., Kamata T., Matsuta S., Katagi M., Miki A., Zaitsu K., Sato T., Nakanishi T., Tsuchihashi H. (2015). Single-hair analysis of zolpidem on the supposition of its single administration in drug-facilitated crimes. Forensic Toxicol..

[74] Kintz P., Villain M., Dumestre-Toulet V., Ludes B. (2005). Drug-facilitated sexual assault and analytical toxicology: The role of LC-MS/MS. A case involving zolpidem. J. Clin. Forensic Med..

[75] Baswan S., Kasting G.B., Li S.K., Wickett R., Adams B., Eurich S., Schamper R. (2017). Understanding the formidable nail barrier: A review of the nail microstructure, composition and diseases. Mycoses.

[76] Cappelle D., Yegles M., Neels H., van Nuijs A., De Doncker M., Maudens K., Covaci A., Crunelle C.L. (2015). Nail analysis for the detection of drugs of abuse and pharmaceuticals: A review. Forensic Toxicol..

[77] Green S.J., Wilson J.F. (1996). The Effect of Hair Color on the Incorporation of Methadone into Hair in the Rat. J. Anal. Toxicol..

[78] Reid R.W., O’Connor F.L., Deakin A.G., Ivery D.M., Crayton J.W. (1996). Cocaine and Metabolites in Human Graying Hair: Pigmentary Relationship. J. Toxicol. Clin. Toxicol..

[79] Palmeri A., Pichini S., Pacifici R., Zuccaro P., Lopez A. (2000). Drugs in Nails. Physiology, pharmacokinetics and forensic toxicology. Clin. Pharmacokinet..

[80] Alexiou D., Koutselinis A., Manolidis C., Boukis D., Papadatos J., Papadatos C. (1980). The Content of Trace Elements (Cu, Zn, Fe, Mg) in Fingernails of Children. Dermatology.

[81] Krumbiegel F., Hastedt M., Tsokos M. (2014). Nails are a potential alternative matrix to hair for drug analysis in general unknown screenings by liquid-chromatography quadrupole time-of-flight mass spectrometry. Forensic Sci. Med. Pathol..

[82] Castillo-Peinado L.S., Luque de Castro M.D. (2017). An overview on forensic analysis devoted to analytical chemists. Talanta.

[83] Malathi N., Mythili S., Vasanthi H.R. (2014). Salivary Diagnostics: A Brief Review. ISRN Dent..

[84] Humphrey S.P., Williamson R.T. (2001). A review of saliva: Normal composition, flow, and function. J. Prosthet. Dent..

[85] Larsen M., Jensen A., Madsen D., Pearce E. (1999). Individual variations of pH, buffer capacity, and concentrations of calcium and phosphate in unstimulated whole saliva. Arch. Oral Biol..

[86] Álvarez-Sánchez B., Priego-Capote F., Luque de Castro M.D. (2012). Study of sample preparation for metabolomic profiling of human saliva by liquid chromatography–time of flight/mass spectrometry. J. Chromatogr. A.

[87] Zhang A., Sun H., Wang X. (2012). Saliva Metabolomics Opens Door to Biomarker Discovery, Disease Diagnosis, and Treatment. Appl. Biochem. Biotechnol..

[88] Mena-Bravo A., Luque de Castro M.D. (2014). Sweat: A sample with limited present applications and promising future in metabolomics. J. Pharm. Biomed. Anal..

[89] Calderón-Santiago M., Priego-Capote F., Jurado-Gámez B., Luque de Castro M. (2014). Optimization study for metabolomics analysis of human sweat by liquid chromatography-tandem mass spectrometry in high resolution mode. J. Chromatogr. A.

[90] Dinis-Oliveira R.J. (2014). Metabolomics of drugs of abuse: A more realistic view of the toxicological complexity. Bioanalysis.

[91] Zhu S.-S., Long R., Song T., Zhang L., Dai Y.-L., Liu S.-W., Zhang P. (2019). UPLC-Q-TOF/MS Based Metabolomics Approach to Study the Hepatotoxicity of Cantharidin on Mice. Chem. Res. Toxicol..

[92] Lin C.-Y., Wu H., Tjeerdema R.S., Viant M.R. (2007). Evaluation of metabolite extraction strategies from tissue samples using NMR metabolomics. Metabolomics.

[93] Harada S., Takebayashi T., Kurihara A., Akiyama M., Suzuki A., Hatakeyama Y., Sugiyama D., Kuwabara K., Takeuchi A., Okamura T. (2015). Metabolomic profiling reveals novel biomarkers of alcohol intake and alcohol-induced liver injury in community-dwelling men. Environ. Health Prev. Med..

[94] Griffin J.L., Scott J., Nicholson J.K. (2007). The Influence of Pharmacogenetics on Fatty Liver Disease in the Wistar and Kyoto Rats: A Combined Transcriptomic and Metabonomic Study. J. Proteome Res..

[95] Griffin J.L., Salek R.M. (2007). Metabolomic applications to neuroscience: More challenges than chances?. Expert Rev. Proteom..

[96] Naz S., Moreira Dos Santos D.C., García A., Barbas C. (2014). Analytical protocols based on LC–MS, GC–MS and CE–MS for nontargeted metabolomics of biological tissues. Bioanalysis.

[97] Gonzalez-Riano C., Garcia A., Barbas C. (2016). Metabolomics studies in brain tissue: A review. J. Pharm. Biomed. Anal..

[98] Kashem M.A., Ahmed S., Sultana N., Ahmed E.U., Pickford R., Rae C., Šerý O., McGregor I.S., Balcar V.J. (2016). Metabolomics of Neurotransmitters and Related Metabolites in Post-Mortem Tissue from the Dorsal and Ventral Striatum of Alcoholic Human Brain. Neurochem. Res..

[99] Huo Z., Yu L., Yang J., Zhu Y., Bennett D.A., Zhao J. (2020). Brain and blood metabolome for Alzheimer’s dementia: Findings from a targeted metabolomics analysis. Neurobiol. Aging.

[100] Thierauf-Emberger A., Echle J., Dacko M., Lange T. (2020). Comparison of ethanol concentrations in the human brain determined by magnetic resonance spectroscopy and serum ethanol concentrations. Int. J. Leg. Med..

[101] Mora-Ortiz M., Trichard M., Oregioni A., Claus S.P. (2019). Thanatometabolomics: Introducing NMR-based metabolomics to identify metabolic biomarkers of the time of death. Metabolomics.

[102] Weljie A.M., Newton J., Mercier P., Carlson E., Slupsky C.M. (2006). Targeted Profiling: Quantitative Analysis of 1H NMR Metabolomics Data. Anal. Chem..

[103] Xiao J.F., Zhou B., Ressom H.W. (2012). Metabolite identification and quantitation in LC-MS/MS-based metabolomics. TrAC Trends Anal. Chem..

[104] Týčová A., Ledvina V., Klepárník K. (2017). Recent advances in CE-MS coupling: Instrumentation, methodology, and applications. Electrophoresis.

[105] Snowden S., Dahlén S.-E., Wheelock C.E. (2012). Application of metabolomics approaches to the study of respiratory diseases. Bioanalysis.

[106] Zhang P., Zhu S., Zhao M., Dai Y., Zhang L., Ding S., Zhao P., Li J. (2018). Integration of 1H NMR- and UPLC-Q-TOF/MS-based plasma metabonomics study to identify diffuse axonal injury biomarkers in rat. Brain Res. Bull..

[107] Worley B. (2012). Multivariate Analysis in Metabolomics. Curr. Metab..

[108] Riaño C.G., González S.T., García A., Muñoz A., DeFelipe J., Barbas C. (2017). Metabolomics and neuroanatomical evaluation of post-mortem changes in the hippocampus. Brain Struct. Funct..

[109] Kang Y.-R., Park Y.S., Park Y.C., Yoon S.M., JongAhn H., Kim G., Kwon S.W. (2012). UPLC/Q-TOF MS based metabolomics approach to post-mortem-interval discrimination: Mass spectrometry based metabolomics approach. J. Pharm. Investig..

[110] Isbell T.A., Strickland E.C., Hitchcock J., McIntire G., Colyer C.L. (2015). Capillary electrophoresis-mass spectrometry determination of morphine and its isobaric glucuronide metabolites. J. Chromatogr. B Anal. Technol. Biomed. Life Sci..

[111] Nielsen K.L., Telving R., Andreasen M.F., Hasselstrøm J.B., Johannsen M. (2016). A Metabolomics Study of Retrospective Forensic Data from Whole Blood Samples of Humans Exposed to 3,4-Methylenedioxymethamphetamine: A New Approach for Identifying Drug Metabolites and Changes in Metabolism Related to Drug Consumption. J. Proteome Res..

[112] Ellis D.I., Dunn W.B., Griffin J.L., Allwood J.W., Goodacre R. (2007). Metabolic fingerprinting as a diagnostic tool. Pharmacogenomics.

[113] Peterson D.S. (2007). Matrix-free methods for laser desorption/ionization mass spectrometry. Mass Spectrom. Rev..

[114] Morelato M., Beavis A., Kirkbride P., Roux C. (2013). Forensic applications of desorption electrospray ionisation mass spectrometry (DESI-MS). Forensic Sci. Int..

[115] Deimler R.E., Razunguzwa T.T., Reschke B.R., Walsh C.M., Powell M.J., Jackson G.P. (2014). Direct analysis of drugs in forensic applications using laser ablation electrospray ionization-tandem mass spectrometry (LAESI-MS/MS). Anal. Methods.

